# Too Young to Suspect, Too Critical to Miss: A Rare Case of Infantile Appendicitis

**DOI:** 10.7759/cureus.110246

**Published:** 2026-06-04

**Authors:** Zoi Lamprinou, Elisavet Kanna, Despina Panayiotou, Eleni Batsari, Ioannis Skondras

**Affiliations:** 1 Pediatric Surgery Department, Panagiotis & Aglaia Kyriakou Childrens' Hospital, Athens, GRC; 2 Otolaryngology Department, General Children's Hospital of Athens "Pan. Aglaia Kyriakou", Athens, GRC; 3 Nursing Department, National and Kapodistrian University of Athens, Athens, GRC

**Keywords:** appendectomy, infant, infantile appendicitis, laparoscopy, peritonitis

## Abstract

Infantile appendicitis is exceptionally rare and difficult to diagnose because of its non-specific presentation. We report the case of a two-month-old male infant presenting with fever, vomiting, diarrhea, abdominal distention, and signs of generalized peritonitis. Diagnostic laparoscopy revealed diffuse purulent peritonitis, a gangrenous perforated appendix, and an intraperitoneal fecalith. Laparoscopic appendectomy and peritoneal lavage were successfully performed without conversion to open surgery. The postoperative course was uneventful. This case highlights the diagnostic challenges of appendicitis in early infancy and demonstrates the feasibility of minimally invasive management even in critically ill young infants.

## Introduction

Appendicitis is one of the most common surgical emergencies in the general pediatric population. However, in neonates and young infants, it is exceedingly rare, affecting less than 2% of children under two years of age [1]. The incidence of appendicitis in the neonatal period is estimated at 0.04-0.2%, with a male predominance of approximately 3:1 [2]. However, cases occurring in the early infantile period, such as our patient at two months of age, remain similarly uncommon.

The condition typically presents with vague symptoms mimicking other common neonatal abdominal pathologies, particularly enterocolitis (necrotizing enterocolitis), gastroenteritis, or simple feeding intolerance [3]. This diagnostic difficulty is compounded by the infant's inability to communicate pain and the limited specificity of both laboratory and imaging findings in this age group.

Furthermore, the pathophysiology of appendicitis in this age group differs significantly from that of older children and adults. Several anatomical and physiological factors contribute to the rapid progression and high rate of perforation observed in this population. The appendiceal wall is remarkably thin with insufficient blood flow, the omentum is poorly developed and unable to wall off infection effectively, and the cecal wall demonstrates low elasticity with correspondingly low resistance to infection [1]. These factors result in perforation rates exceeding 80% in neonatal appendicitis, which is substantially higher than the approximately 30% perforation rate observed in older children [4]. When perforation occurs, the consequences are severe, with mortality rates historically reaching as high as 78% in older literature, although more recent reports document rates between 8% and 28% [4]. The introduction of advanced diagnostic modalities, improved perioperative care, and enhanced neonatal intensive care support has contributed to improved survival, yet the condition remains life-threatening.

The role of laparoscopy in neonatal surgical emergencies has evolved considerably over the past two decades. Early concerns regarding the feasibility and safety of minimally invasive approaches in critically ill neonates have been tempered by accumulating experience demonstrating the benefits of laparoscopy as both a diagnostic and therapeutic tool [5]. In cases of acute neonatal abdomen, diagnostic laparoscopy enables direct visualization of intra-abdominal structures, allows identification of the underlying pathology, and permits immediate therapeutic intervention when appropriate while avoiding unnecessary laparotomy in approximately 53% of cases [5]. We report a case of successful laparoscopic management of neonatal appendicitis with generalized peritonitis in a two-month-old male, highlighting the diagnostic approach, operative findings, and postoperative management that led to successful patient outcomes.

## Case presentation

A two-month-old male infant, born at term via caesarean section following an uncomplicated pregnancy and perinatal course, presented with a six-hour history of fever, increasing irritability, and 10 episodes of vomiting. He had been feeding well prior to the onset of symptoms but had not passed stool for the preceding two days. He had no significant past medical history, took no regular medications, and his prenatal and neonatal courses were unremarkable. He was discharged home from the newborn nursery in good condition at 48 hours of life.

On initial examination, the infant appeared alert but irritable. Vital signs revealed a temperature of 37.6°C, a heart rate of 122 beats per minute, a respiratory rate of 35 breaths per minute, and a blood pressure of 78/38 mmHg. Oxygen saturation was 98% on room air. Abdominal examination revealed mild distention and generalized abdominal tenderness on palpation. There was no palpable mass. Inguinal examination revealed no evidence of a hernia. The remainder of the physical examination was unremarkable.

Initial laboratory evaluation demonstrated elevated procalcitonin and lactate levels despite a normal white blood cell count and CRP. Follow-up testing showed a marked inflammatory response with rising white blood cell count and CRP levels (Table 1). Initial abdominal radiography showed no evidence of bowel obstruction or pneumoperitoneum. Abdominal ultrasound excluded pyloric stenosis and intussusception, demonstrating only a small amount of free fluid and a few enlarged mesenteric lymph nodes.

**Table 1:** Laboratory findings during hospitalization, including initial and follow-up inflammatory markers and metabolic parameters, with corresponding reference ranges.

Parameter	Initial value	Follow-up value	Reference range
White blood cell count	5.5 × 10⁹/L	11.7 × 10⁹/L	5-19.5 × 10⁹/L
C-reactive protein (CRP)	1 mg/L	97 mg/L ↑	<5 mg/L
Procalcitonin	14.6 ng/mL ↑	-	<0.5 ng/mL
pH	7.39	-	7.35-7.45
Base deficit	−6.9 mEq/L ↓	-	−2 to +2 mEq/L
Lactate	4.8 mmol/L ↑	-	0.5-2.2 mmol/L

Following admission, the infant developed diarrhea with persistent fever, raising suspicion for gastroenteritis. Stool cultures were obtained and intravenous cefotaxime was initiated. Although vomiting resolved and oral feeding was tolerated, inflammatory markers continued to rise, prompting the addition of metronidazole. Over the next two days, the patient remained clinically stable with a slight decline in inflammatory markers, although the fever and diarrhea persisted. Stool, urine, and cerebrospinal fluid cultures returned negative. A repeat abdominal ultrasound was performed, revealing findings suggestive of perforated appendicitis. The presence of free anechoic fluid in the abdominal cavity further raised concern for peritonitis. The patient was therefore considered to require urgent surgical intervention.

**Figure 1:**
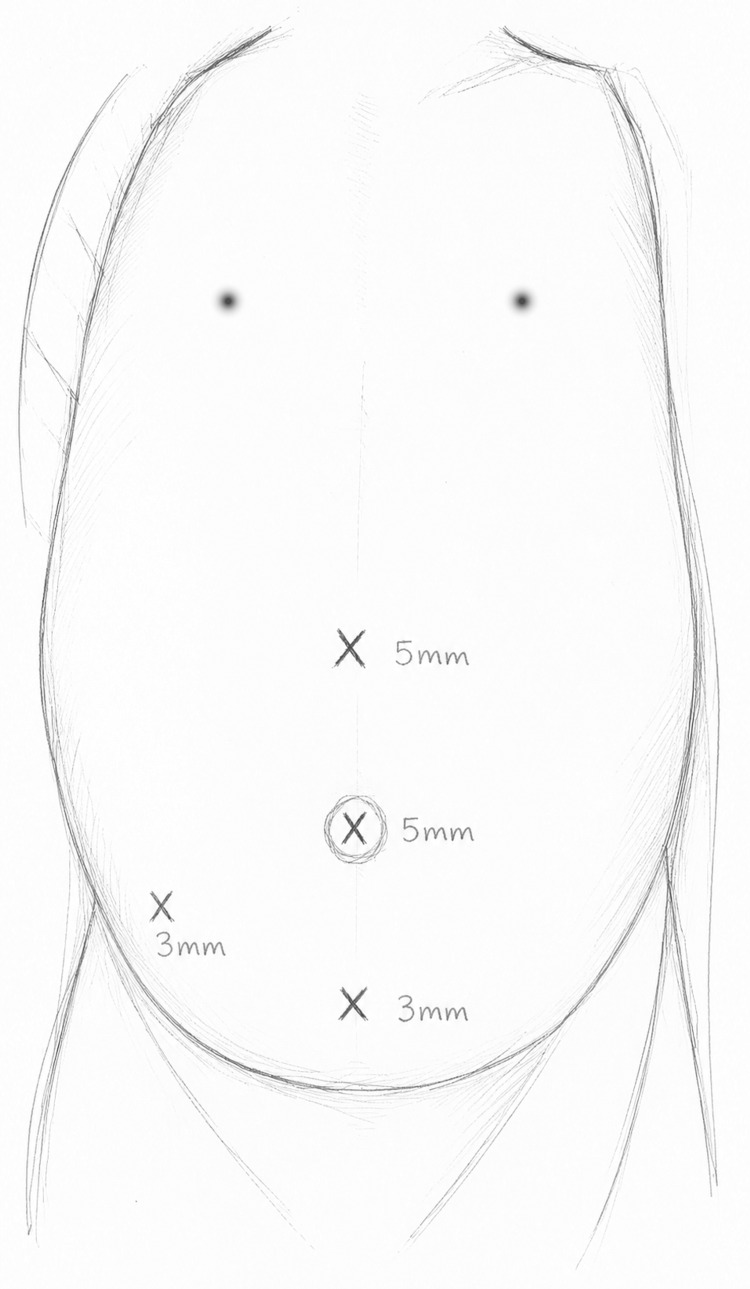
Schematic illustration of trocar placement during laparoscopic appendectomy. Two 5 mm trocars were placed at the umbilical and epigastric positions, while two 3 mm trocars were inserted at the hypogastric and right lower quadrant positions.

The patient was transferred to the operating room, where two 5 mm trocars (umbilical and epigastric) and two 3 mm trocars (hypogastric and right lower quadrant) were inserted using an open technique to establish pneumoperitoneum at low insufflation pressures (4-6 mmHg). The abdominal cavity was carefully inspected systematically. Intraoperative findings confirmed the suspicion of appendicitis. The appendiceal wall appeared gangrenous with areas of full-thickness inflammation. The fourth trocar was required to allow the introduction of a suction-irrigation device because of the significant intra-abdominal inflammatory fluid. A schematic representation of trocar placement and port configuration is provided in Figure 1.

**Figure 2:**
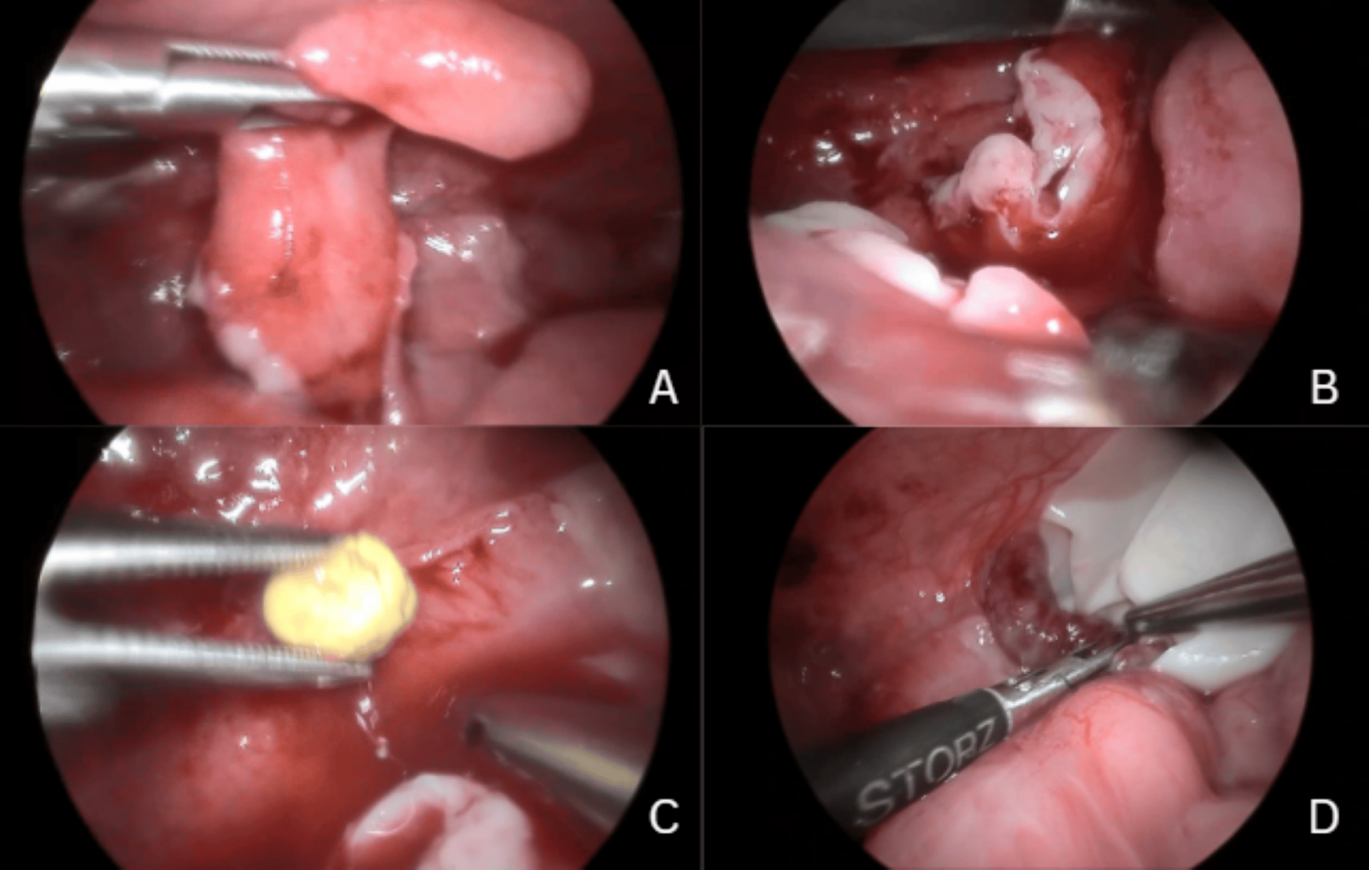
Laparoscopic intraoperative findings during appendectomy. (A) Inflamed and gangrenous appendix with marked edema. (B) Perforation identified at the appendiceal base with surrounding inflammatory changes and purulent contamination. (C) Intraoperative retrieval of an appendicolith from the abdominal cavity. (D) Placement of the resected appendix into an extraction glove prior to removal through the trocar site to minimize intra-abdominal and wound contamination.

Critical to the diagnosis, a small perforation was identified at the appendiceal base (Figure 2Α and Β) with purulent material and appendicolith evident within the abdominal cavity (Figure 2C). The peritoneal surfaces demonstrated fibrinous exudate consistent with acute peritonitis. Loculated collections of purulent fluid were identified in the right upper and lower quadrants, consistent with generalized peritonitis. The small bowel and colon were examined systematically and appeared viable with no other apparent pathology. Laparoscopic appendectomy was successfully performed. The appendix was retrieved using an improvised extraction glove to minimize intra-abdominal and wound contamination (Figure 2D).

Given the perforation at the base of the appendix, an additional intracorporeal suture was placed to securely invaginate and reinforce the appendiceal stump.The peritoneal cavity was irrigated thoroughly with warm normal saline solution until the irrigant cleared, removing purulent material and fibrinous exudate. A drain was placed near the appendectomy site and exteriorized through the right lower quadrant trocar site. The procedure was completed laparoscopically without the need for conversion to an open approach. Total operative time was 105 minutes, and blood loss was minimal.

Following surgery, the patient received intravenous cefotaxime, amikacin, and metronidazole for a total of 10 days. This antibiotic regimen was chosen to ensure broad-spectrum coverage against gram-positive, gram-negative, and anaerobic organisms commonly implicated in complicated intra-abdominal infections [1]. Inflammatory markers declined appropriately, and the patient remained afebrile throughout the early postoperative period, with the exception of a single fever episode of 38.2°C on postoperative day 5 following an attempted discontinuation of intravenous antibiotics.

The patient was kept nil per os for the first 48 hours after surgery. Enteral feeding was then cautiously initiated on postoperative day 2 with small trophic volumes of formula, which were gradually advanced as tolerated. He was discharged on postoperative day 10 in good condition, tolerating full feeds, and with normal vital signs and physical examination findings. Follow-up visits were scheduled with pediatric surgery at 2 and 4 weeks post-discharge, during which the patient demonstrated normal growth and age-appropriate development, with no complications attributable to the appendicitis or the surgical procedure.

## Discussion

The diagnosis of appendicitis in a two-month-old infant is highly challenging due to the rarity of the condition at this age. Symptoms such as vomiting, fever, and diarrhea overlap with more common infantile conditions, including enterocolitis, gastroenteritis, and intussusception [3]. Abdominal distention is the most frequent presenting symptom (64-89%), while vomiting occurs in 47-54% of cases [3]. Fever and diarrhea are also common, although fever may be absent in up to 50% of neonates because of immature immune responses.

A high index of suspicion is crucial for timely diagnosis. A recent review reported a preoperative diagnostic rate of only 17% over a 30-year period [3], with most cases diagnosed intraoperatively or at autopsy. Early imaging, including abdominal ultrasound and, when necessary, computed tomography, can facilitate earlier recognition of intra-abdominal inflammation [6].

Traditionally, neonatal and infantile appendicitis has been managed with open appendectomy via exploratory laparotomy, largely because perforation and complex intra-abdominal findings are common [3]. However, growing experience with diagnostic laparoscopy has shown important advantages. A systematic review found that laparoscopy avoided unnecessary laparotomy in 53% of neonatal acute abdomen cases and enabled definitive laparoscopic treatment in 41% [5]. Benefits include reduced surgical trauma, less postoperative pain, faster recovery of bowel function, and better visualization of the abdominal cavity [3]. In our case, the appendix was retrieved using an extraction glove, a technique associated with lower morbidity and improved surgical outcomes [7].

Despite advances in management, infantile appendicitis may still have severe outcomes. Mortality has decreased from 78% in the early 20th century to 8-28% in recent decades, likely due to improvements in imaging, perioperative care, and neonatal intensive care [4]. A systematic review of infants up to three months old reported mortality in 8% and morbidity in another 8% of patients, with all fatal cases involving abdominal appendicitis [3]. Delayed or missed diagnosis remains particularly dangerous. Recent reports describe fatal cases initially misdiagnosed as gastroenteritis, leading to delayed surgery and septic shock [8]. These cases highlight the importance of maintaining appendicitis in the differential diagnosis of neonates and young infants with unexplained abdominal symptoms and ensuring prompt imaging and close follow-up when the diagnosis is uncertain.

## Conclusions

Infantile appendicitis remains an exceedingly rare yet life-threatening condition that continues to challenge clinicians across multiple specialties. The successful application of laparoscopic appendectomy in this two-month-old patient adds to the growing body of literature supporting minimally invasive approaches from pediatric surgeons with appropriate laparoscopic expertise and anesthetic support as an initial operative strategy, with the understanding that conversion to open surgery is always an option if technical or clinical circumstances warrant.
